# In Silico Analysis of the Molecular Interaction between Anthocyanase, Peroxidase and Polyphenol Oxidase with Anthocyanins Found in Cranberries

**DOI:** 10.3390/ijms251910437

**Published:** 2024-09-27

**Authors:** Victoria Araya, Marcell Gatica, Elena Uribe, Juan Román

**Affiliations:** Laboratorio Enzimología, Departamento de Bioquímica y Biología Molecular, Facultad de Ciencias Biológicas, Universidad de Concepción, Concepción 4070409, Chile; victoriaraya@udec.cl (V.A.); marcgatica@udec.cl (M.G.); auribe@udec.cl (E.U.)

**Keywords:** cranberries, anthocyanase, peroxidase, polyphenol oxidase, anthocyanins degradation, molecular docking, molecular dynamics

## Abstract

Anthocyanins are bioactive compounds responsible for various physiological processes in plants and provide characteristic colors to fruits and flowers. Their biosynthetic pathway is well understood; however, the enzymatic degradation mechanism is less explored. Anthocyanase (β-glucosidase (BGL)), peroxidase (POD), and polyphenol oxidase (PPO) are enzymes involved in degrading anthocyanins in plants such as petunias, eggplants, and *Sicilian* oranges. The aim of this work was to investigate the physicochemical interactions between these enzymes and the identified anthocyanins (via UPLC-MS/MS) in cranberry (*Vaccinium macrocarpon*) through molecular docking to identify the residues likely involved in anthocyanin degradation. Three-dimensional models were constructed using the AlphaFold2 server based on consensus sequences specific to each enzyme. The models with the highest confidence scores (pLDDT) were selected, with BGL, POD, and PPO achieving scores of 87.6, 94.8, and 84.1, respectively. These models were then refined using molecular dynamics for 100 ns. Additionally, UPLC-MS/MS analysis identified various flavonoids in cranberries, including cyanidin, delphinidin, procyanidin B2 and B4, petunidin, pelargonidin, peonidin, and malvidin, providing important experimental data to support the study. Molecular docking simulations revealed the most stable interactions between anthocyanase and the anthocyanins cyanidin 3-arabinoside and cyanidin 3-glucoside, with a favorable ΔG of interaction between −9.3 and −9.2 kcal/mol. This study contributes to proposing a degradation mechanism and seeking inhibitors to prevent fruit discoloration.

## 1. Introduction

In nature, plants suffer various threats that can affect their survival, including extreme environmental events, changes in temperatures, and rainfall patterns because of climate change [1]. Plants respond to stress by the activation of specific molecular and physiological responses [2]. Secondary metabolites play an essential role as a defense mechanism against biotic and abiotic stressors such as pathogens, herbivores, extreme temperatures, drought, and high levels of UV radiation [3]. These compounds allow plants to adapt and survive in dynamic environments, including those caused by climate change. Anthocyanins are a type of flavonoid pigment that can be found in various fruits and vegetables, imparting colors ranging from pale yellow to red, purple, and blue, with the latter being most prevalent in berries, flowers, and fruits. These pigments serve as a visual signal that attracts pollinators for seed dispersal [4,5].

Anthocyanins are found in a wide variety of vegetable and fruit families, including *Rosaceae*, *Ericaceae*, *Vitaceae*, *Brassicaceae*, *Solanaceae,* and *Leguminosae*. These metabolites are essential for the development of plant organs and tissues, as well as adaptation to biotic and abiotic stress [6,7,8].

**Figure 1:**
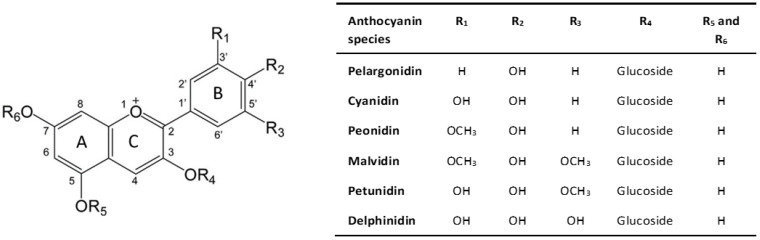
Chemical structure of anthocyanin. Adapted from [12].

Anthocyanins, commonly known as anthocyanidin glycosides, belong to the flavonoid family and exhibit a basic structure comprising an anthocyanidin chromophore, derived from the 2-phenylbenzopyrilium (flavylium) skeleton. This structure includes two benzoic rings (rings A and B) and a heterocyclic ring (ring C), forming a fundamental C6-C3-C6 skeleton [9], as shown in Figure 1.

The chemical diversity of anthocyanins, with over 700 types identified in nature, is attributed to variations in the rings, particularly in the number of hydroxyl and methoxyl groups attached to ring B [10]. Six anthocyanidins, including cyanidin, delphinidin, pelargonidin, peonidin, petunidin, and malvidin, are most prevalent in foods, differing in the number of hydroxyl and methoxyl groups at the R_1_ and R_2_ positions, while sharing a common hydroxylation pattern at the 3, 5, and 7 positions [11].

The addition of a sugar chain results in the glycosidic form of the anthocyanidin molecule, known as anthocyanin [9].

Plants rich in anthocyanins have multiple benefits for human health, such as protection against heart, diabetic, visual, and cognitive diseases, and have anti-inflammatory, anticancer, and antitumor effects [13]. These molecules have a high antioxidant activity against hydrogen peroxide (H_2_O_2_), peroxide radicals (ROO·), superoxide (·O_2_^−^), hydroxyl (·OH), and singlet oxygen (^1^O_2_) [14]. Despite the multiple benefits of anthocyanins, their chemical instability and tendency to degrade easily represent two of the main limitations in the food industry. This can negatively affect the molecule’s bioavailability and the nutritional quality of foods.

Cranberry (*Vaccinium macrocarpon*) is a small evergreen shrub belonging to the genus *Vaccinium*, family *Ericaceae*, found mainly in temperate and cold regions of North and South America [15]. This fruit is a rich source of antioxidants like anthocyanins and polyphenols, containing up to 200–300 mg polyphenols per 100 g fresh weight [16,17]. The color spectrum of cranberry fruits, ranging from red to dark red or dark purple, is predominantly due to the presence of anthocyanins. Studies have identified six primary anthocyanins in cranberries: cyanidin-3-galactoside, cyanidin-3-glucoside, cyanidin-3-arabinoside, peonidin-3-galactoside, peonidin-3-glucoside, and peonidin-3-arabinoside [18,19,20,21,22,23,24]. The diverse composition of anthocyanins in cranberries significantly contributes to their distinctive color, as well as their remarkable nutritional and medicinal qualities. These properties are linked to a range of bioactivities, such as inhibiting bacterial adhesion and providing bacteriostatic and anti-inflammatory effects. Additionally, they offer support for urinary tract, cardiovascular, oral, and gastrointestinal health [25,26,27,28].

The primary challenge with fruits like cranberries lies in their high perishability and the chemical instability of anthocyanins, which are easily degraded. The concentration of anthocyanins in cranberries is influenced by various environmental factors such as geographical location, temperature, maturity, and exposure to light and heat during transportation and storage [29,30,31,32]. Additionally, oxidation during processing, improper ripening during harvesting, and enzymatic degradation significantly contribute to the degradation of anthocyanins [15]. While most studies have focused on harvest and postharvest processing conditions, addressing external factors, research on the enzymatic degradation of anthocyanins in cranberries remains limited.

Anthocyanin degradation by endogenous enzymes, such as hydrolyzing, oxidizing, or decarboxylating enzymes, leads to the loss of color and antioxidant activity. This enzymatic degradation, beneficial for plants in certain contexts such as pollination, can be triggered by factors like insufficient light exposure and temperature [31,33,34].

**Figure 2:**
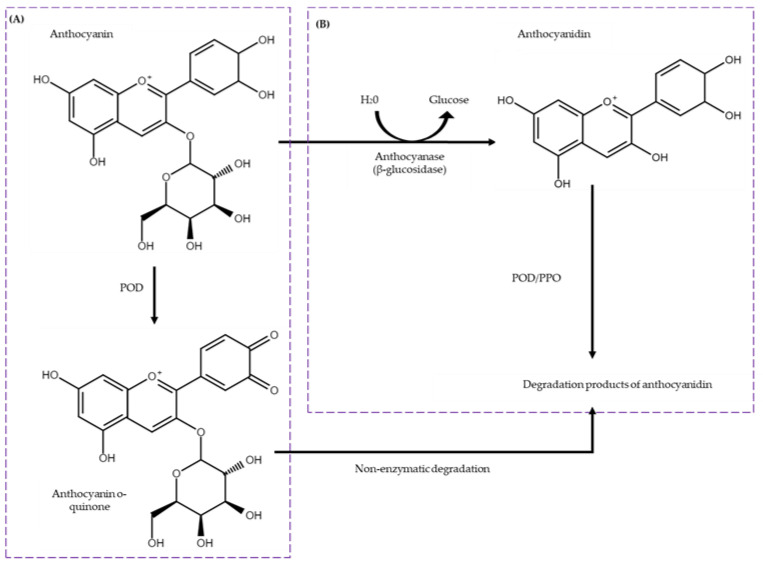
Enzymatic degradation pathways of anthocyanins (Cyanidin-3-O-glucoside). The mechanism includes two distinct pathways: (**A**) the direct oxidation of anthocyanins by peroxidase (POD) and (**B**) a two-step degradation process beginning with the cleavage of the glycosidic bond by β-glucosidase (BGL) followed by oxidation through polyphenol oxidase or peroxidase (PPO/POD). Adapted from [35,39].

It has been postulated that three enzyme families are involved in the degradation of anthocyanins: anthocyanase (β-glucosidase (BGL)), polyphenol oxidase (PPO), and class III peroxidase (POD) (Figure 2). BGL is responsible for the breakdown of anthocyanins, hydrolyzing the glycosidic bonds of anthocyanins to produce free sugar and aglycone. On the other hand, PPO and POD are enzymes that would catalyze the oxidation of anthocyanins, leading to a loss of color and flavor [32,35,36,37,38]. Figure 2 specifically illustrates the degradation pathways of cyanidin-3-O-glucoside. While other anthocyanins with different sugar moieties, such as galactose or arabinose, are present in cranberries, cyanidin-3-O-glucoside provides a detailed exploration of a commonly occurring degradation mechanism. The transformation of anthocyanin o-quinone to degradation products of anthocyanidin is depicted, consistent with the enzymatic degradation processes described in the literature [35,36,37,38].

Despite this understanding, the exact process of anthocyanin degradation in most vegetables remains unclear, with insights from studies on fruit extracts and juices suggesting a complex system controlling pigment concentration in plants. Investigating these enzymatic mechanisms could be key to preserving the nutritional quality and health benefits of such fruits.

## 2. Results

### 2.1. Identification of Anthocyanins in Cranberries Using UPLC-MS/MS Analysis

In this investigation, Ultra-Performance Liquid Chromatography coupled with Tandem Mass Spectrometry (UPLC-MS/MS) was employed to identify anthocyanins present in cranberry samples. Fourteen distinct flavonoids, including anthocyanins and related compounds were detected, namely Cyanidin, Cyanidin-3-galactoside, Delphinidin, Procyanidin B4, Procyanidin B2, Petunidin, Pelargonidin, Peonidin, Malvidin, Rutin, Luteolin, Quercetin, Kaempferol, and Isorhamnetin. Detailed results, including their retention times, mass-to-charge ratios (*m*/*z*), and concentrations, are presented in Table 1 (Figure S1).

These anthocyanins and related compounds can be categorized based on their aglycone and sugar moieties [40]. It is important to note the structural differences between anthocyanidins and other flavonoids. While all anthocyanins are flavonoids, not all flavonoids are anthocyanins. For instance, quercetin is a flavonol and luteolin is a flavone. Cyanidin-based anthocyanins, including Cyanidin-3-galactoside, are known for their potent antioxidant properties [13]. Cyanidin compounds are characterized by the presence of hydroxyl groups on the B-ring of the flavilium ion, contributing to their red coloration and health benefits [9,13]. Delphinidin and Petunidin are characterized by additional hydroxyl groups, which enhance their antioxidant activity and impart blue to purple hues [9,41]. On the other hand, Peonidin-based anthocyanins, such as Peonidin, contain a methoxyl group on the B-ring, which imparts distinct stability and hue [41]. Pelargonidin- and Malvidin-based anthocyanins exhibit variations in their hydroxylation and methylation patterns, affecting their color and stability [9,42].

Other identified compounds, such as Procyanidin B2 and Procyanidin B4, are flavanols known for their antioxidant properties and contributions to the astringency of cranberries [43,44]. Rutin, Luteolin, Quercetin, Kaempferol, and Isorhamnetin are flavonoids with various health benefits, including anti-inflammatory and anti-carcinogenic properties [45].

### 2.2. Structural Modeling of Anthocyanin-Degrading Enzymes in Cranberries

In this study, we utilized computational modeling to investigate the structural dynamics of enzyme–substrate interactions in three enzymes involved in anthocyanin degradation in cranberries: β-glucosidase (BGL), peroxidase (POD), and polyphenol oxidase (PPO). Due to the lack of crystallographic structures for these enzymes in cranberry, three-dimensional models were constructed using the AlphaFold2 server, based on the consensus sequences specific to each enzyme.

**Figure 3:**
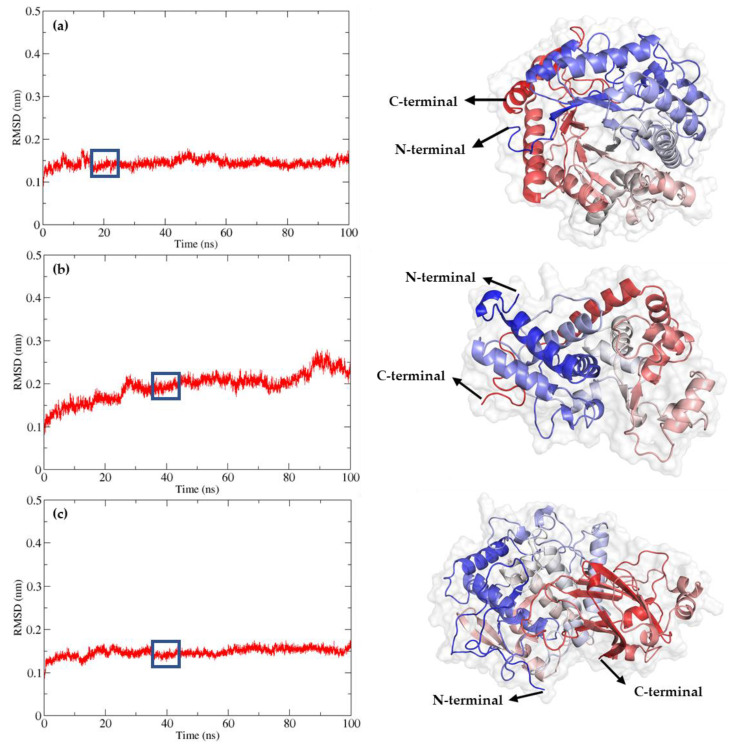
Refinement of AlphaFold2-generated models through 100 ns molecular dynamic simulations. Root Mean Square Deviation (RMSD) and refined 3D model of (**a**) BGL, (**b**) POD, and (**c**) PPO. The models were visualized using PyMOL, and the colors from the N-terminal to the C-terminal were based on their secondary structure.

Among the generated models, those with the highest predicted Local Distance Difference Test (pLDDT) scores and pTM scores above 0.5 were selected for further analysis. The BGL model exhibited a pLDDT score of 87.6 and a pTM score of 0.857, displaying a (β/α) eight-barrel topology comprising 20 alpha-helices, 13 beta-sheets, and 34 loops. The POD model showed a pLDDT of 94.8 and a pTM of 0.914 with 19 alpha-helices, two beta-sheets, and 21 loops. Similarly, the PPO model had a pLDDT of 84.1 and a pTM of 0.831, featuring 22 alpha-helices, 11 beta-sheets, and 32 loops (Figure 3).

To evaluate the stability and dynamic properties of these models, molecular dynamics (MD) simulations were conducted for 100 nanoseconds (ns). The Root Mean Square Deviation (RMSD) values of the backbone atoms were monitored to assess equilibrium state and structural stability. For the BGL enzyme, RMSD values stabilized after 18 ns, with an average RMSD of 0.15 nm, indicating robust structural integrity throughout the simulation, varying between 0.14 and 0.16 nm. The final model was obtained around 20 ns. For the POD enzyme, RMSD values stabilized after 30 ns, with an average RMSD of 0.18 nm, varying between 0.13 and 0.20 nm. The final model was obtained around 40 ns. For the PPO enzyme, RMSD values stabilized after 20 ns, with an average RMSD of 0.15 nm, varying between 0.13 and 0.16 nm. The final model was obtained around 40 ns.

Figure 3 provides a detailed visualization of the refined 3D models of BGL, POD, and PPO, following the 100 ns MD simulations. The models were rendered using PyMOL, with color gradations from the N-terminal to the C-terminal based on their secondary structures. The refined models of BGL, POD, and PPO are available in the Protein Model Database (PMDB) with the respective identifiers PM0084604 for BGL, PM0084605 for POD, and PM0084606 for PPO.

### 2.3. Molecular Interaction Analysis

In this study, the interaction between identified anthocyanins and key enzymes involved in their degradation was investigated through molecular docking simulations. The enzymes β-glucosidase (BGL), peroxidase (POD), and polyphenol oxidase (PPO) were modeled and their interactions with anthocyanins were analyzed. These interactions highlight the potential pathways for the enzymatic degradation of anthocyanins in cranberries, providing a deeper understanding of the molecular mechanisms involved.

Table 2 presents the calculated ΔG binding derived from molecular docking simulations between β-glucosidase (BGL), peroxidase (POD), and polyphenol oxidase (PPO) with various anthocyanins and related compounds identified in cranberries. Established positive controls, specifically sucrose (SUC) for BGL, epicatechin (EPC) for POD, and 3,4-dihydroxyphenylacetic acid (3,4-DHPA) for PPO, were used as reference points to benchmark the enzymatic affinities. The selection of reference compounds for interaction studies with BGL, POD, and PPO was based on their established binding affinities and common usage in enzymatic assays. These compounds were chosen to ensure reliable and relevant docking simulations. The focus is on specific residues within each enzyme’s active site involved in substrate interaction, providing a comprehensive understanding of the molecular mechanisms supporting these enzymatic processes.

Molecular docking simulations revealed variations in the ΔG binding among the enzymes and the identified cranberry anthocyanins. Cyanidin-3-arabinoside and Cyanidin-3-glucoside exhibited high binding energies, indicating strong interactions with BGL, POD, and PPO. For BGL, key residues such as Gln88, His377, and Gly453 were involved in substrate recognition and binding, suggesting potential enzymatic pathways for anthocyanin degradation.

On average, the ΔG binding energies were highest for BGL at −8.8 kcal/mol, followed by POD at −8.1 kcal/mol and PPO at −6.0 kcal/mol. Among the various anthocyanins and related compounds, BGL showed the best docking results, with a maximum binding energy of −10.1 kcal/mol for Rutin.

The docking studies also included an analysis of 27 flavonoids identified in *Vaccinium* species, 19 of which are anthocyanins. These values are consistent with the previously published literature describing Cyanidin and Peonidin derivatives as the major anthocyanins in cranberries.

Molecular docking studies of the anthocyanins and related compounds showed that in each docking scenario, there was an interaction with either glutamic acid or aspartic acid residues. The most stable interaction was between BGL and Rutin, involving key residues such as His189, Glu451, and Gly453. The next most stable interaction was between BGL and Cyanidin-3-arabinoside, with residues Gln88, Asn234, Glu451, His377, and Gly453 participating.

The molecular docking studies indicated that the most stable interaction for POD was with Rutin, involving key residues such as Arg53, His57, Asn147, Lys187, and Asp225, all within 3 Å of the Rutin. The highest affinity ligand for POD was Rutin with a binding energy of −8.9 kcal/mol. The binding energy range for POD-substrate dockings was between −8.9 and −7.7 kcal/mol.

For PPO, the most stable interaction was with Cyanidin-3-arabinoside, involving key residues like Lys502, Asp319, Asn533, His330, and Val534, all within 3 Å of the Cyanidin-3-arabinoside. The highest affinity ligand for PPO was Luteolin, with a binding energy of −8.6 kcal/mol. The binding energy range for PPO-substrate dockings was between −8.6 and −3.8 kcal/mol.

These molecular docking studies provide a detailed understanding of the interactions between the enzymes BGL, POD, and PPO, and the major anthocyanins and related compounds in cranberries, offering insights into their potential enzymatic degradation pathways.

**Figure 4:**
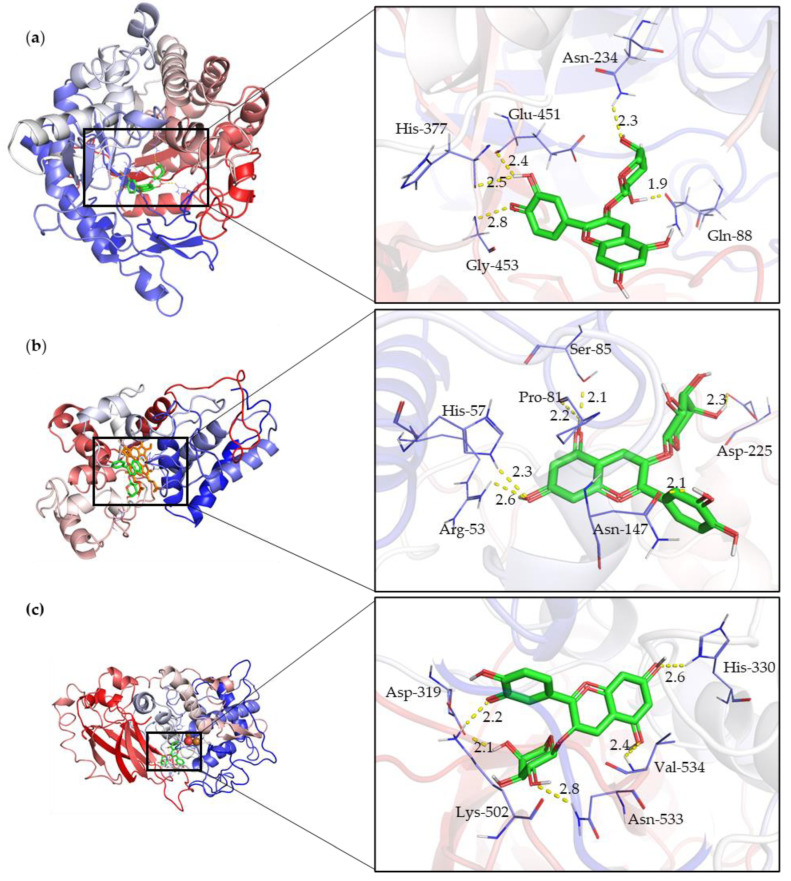
Molecular interactions of Cyanidin 3-Arabinoside (depicted in green) with enzymes (**a**) β-glucosidase (BGL), (**b**) Peroxidase (POD), and (**c**) Polyphenol oxidase (PPO), highlighting the interaction of the active sites of each enzyme with the ligand.

Figure 4 illustrates the molecular interactions of Cyanidin 3-Arabinoside (depicted in green) with the enzymes β-glucosidase (BGL), peroxidase (POD), and polyphenol oxidase (PPO), highlighting the interaction of the active sites of each enzyme with the ligand. Cyanidin 3-Arabinoside is shown as it demonstrated the most favorable binding energy among the docking results, serving as an exemplary anthocyanin in this context.

## 3. Discussion

In this study, UPLC-MS/MS analysis was conducted to identify anthocyanins in cranberries. By comparing our findings with the existing literature, several observations can be made. For cyanidin-based anthocyanins, our study showed that Cyanidin-3,5-diglucoside was not detected (n.d.), which aligns with findings that concentrations can vary widely depending on factors such as ripening stage and processing methods [43]. Cyanidin-3-galactoside was detected at a high concentration of 27.687 mg/(100 g dm), which is lower than the 119.9–180.0 mg/(100 g dm) range reported in other studies using LC/MS Q-TOF and UPLC-PDA-FL [19,20]. Cyanidin itself was found at 0.005 mg/(100 g dm) in our study. Other studies have reported that Cyanidin derivatives range from 442 to 967 mg/(100 g dm), detected using LC/MS Q-TOF and UPLC-PDA-FL [46]. Other authors have reported that Cyanidin-3-O-arabinoside ranges from 64.5 to 95.6 mg/(100 g dm) [19,20] and Cyanidin-3-O-glucoside ranges from 5.5 to 7.3 mg/(100 g dm), using the same techniques [19,20].

For Delphinidin and Petunidin, our study detected Delphinidin at 0.025 mg/(100 g dm). Other studies have reported that Delphinidin derivatives range from 31.27 to 43.87 mg/(100 g dm), detected using LC/MS Q-TOF and UPLC-PDA-FL [46]. Delphinidin-3-O-glucoside is found at concentrations between 1.1 and 1.8 mg/(100 g dm) using the same techniques [19]. It is noted that Delphinidin is present in cranberries but usually in lower concentrations compared to cyanidin-based anthocyanins, which is consistent with our findings. Petunidin was detected at 0.003 mg/(100 g dm), and its presence in cranberries is noted, although typically in lower concentrations. In a recent study, Petunidin derivatives in cranberries were found to be within a similar range, supporting our findings [41].

Regarding procyanidins, Procyanidin B4 was detected at 0.277 mg/(100 g dm). It is suggested that Procyanidin B4 contributes to the astringency and antioxidant properties of cranberries, which is consistent with our findings. Procyanidin B2 was detected at a much higher concentration of 2.034 mg/(100 g dm). This aligns with findings that highlight Procyanidin B2 as a significant contributor to the antioxidant capacity of cranberries [44,47]. Other authors report that polymeric proanthocyanidins were found in concentrations ranging from 651 to 1109 mg/(100 g dm), detected using LC/MS Q-TOF and UPLC-PDA-FL [46].

In our study, Pelargonidin- and Malvidin-based anthocyanins showed the lowest concentrations compared to the other molecules reported (Table 1). Pelargonidin was detected at 0.002 mg/(100 g dm), and Malvidin was found at 0.001 mg/(100 g dm). Other studies have reported that Malvidin derivatives range from 29.85 to 58.85 mg/(100 g dm), detected using LC/MS Q-TOF and UPLC-PDA-FL [46]. Malvidin-3-O-arabinoside is found at concentrations between 1.4 and 1.9 mg/(100 g dm) using the same techniques [46]. This aligns with our findings that Malvidin is present in cranberries, but typically in low concentrations.

Peonidin-based anthocyanins, such as Peonidin, were detected at 0.004 mg/(100 g dm). Peonidin’s role in the stability and color of cranberry anthocyanins is highlighted, which is consistent with our data. Other studies have reported that Peonidin derivatives range from 192 to 666 mg/(100 g dm), detected using LC/MS Q-TOF and UPLC-PDA-FL [46]. Specifically, Peonidin-3-O-galactoside has been found at concentrations between 131.3 and 310.3 mg/(100 g dm) [19,20,48]. Additionally, Peonidin-3-O-arabinoside ranges from 42.9 to 95.2 mg/(100 g dm) [20,48]. These findings support the significant presence of Peonidin derivatives in cranberries, aligning with our data.

Other flavonoids, including Rutin, Luteolin, Quercetin, Kaempferol, and Isorhamnetin, were detected in various concentrations, with Quercetin being the most abundant at 0.452 mg/(100 g dm). The health benefits of these flavonoids, emphasizing their anti-inflammatory and anti-carcinogenic properties, support their significant presence in our study [43].

In plants, anthocyanins play several roles, such as in pollination and defense, and their presence or absence is determined by the balance between biosynthesis and degradation [4,5]. While their biosynthetic pathway has been well studied [49,50,51,52], knowledge about the enzymatic degradation mechanism is limited. BGL, PPO, and POD enzymes have been suggested to be involved in anthocyanin degradation. In plants like petunias and fruits, such as eggplant and *Sicilian orange*, anthocyanase (β-glucosidase) has been identified as the enzyme responsible for the enzymatic degradation of anthocyanins [35,39].

In addition to identifying the anthocyanins present in cranberries, this study explored the enzymatic pathways responsible for their degradation. Our molecular docking simulations indicated that BGL, PPO, and POD enzymes interact with anthocyanins at specific active site residues, facilitating their breakdown. These findings align with known enzymatic mechanisms in other plant systems, providing a broader context for understanding anthocyanin stability and degradation in cranberries. This detailed understanding of enzymatic interactions and pathways underscores the importance of these enzymes in modulating the anthocyanin content in cranberries, thereby influencing their nutritional and sensory qualities.

Following the analysis of anthocyanins, this study proceeded to the structural modeling of anthocyanin-degrading enzymes in cranberries, focusing on β-glucosidase (BGL). From a structural perspective, BGL comprises two major loops: the helix alpha loop and the helix beta loop. This overall structure indicates a classical barrel domain (β/α)8, known as the TIM barrel, which is commonly observed in members of the glucoside hydrolase family 1 (GH1) [53,54]. Using the INTERPRO server, it was determined that the modeled BGL belongs to the GH1 family, characterized by a catalytic site with two acidic glutamic residues located at the C-terminal ends of beta sheets 4 and 7, specifically in the TENEP and TENG domains [55,56] (http://www.cazy.org/GH1.html accessed on 1 March 2024). In the modeled BGL, these domains were present, with the putative catalytic residues identified as GLU235 and GLU451. Based on homology, it is expected that the underlying mechanism is similar to other retaining glucoside hydrolases from GH1 [57,58].

The retention mechanism of BGL enzymes involves a pair of acid/base and nucleophilic residues positioned on opposite sides of the sugar, separated by a distance of less than 5 Å, as observed in the modeled BGL. The remaining BGL enzymes perform catalysis in two steps: glycosylation and deglycosylation [55].

In terms of molecular docking, the hydrolysis reaction is facilitated by two amino acid residues (typically glutamic acid) separated by approximately 5 Å, functioning as nucleophiles and proton donors, respectively. In the first step of the catalytic mechanism, Glu235 acts as an acid donating a proton to the leaving group, while the catalytic nucleophile (Glu451) attacks from the opposite end to form a glycosyl-enzyme intermediate. In the second step, the catalytic base (Glu235) extracts a proton from a water molecule, increasing its nucleophilicity to attack the anomeric carbon and displace the enzyme [55].

Studies of molecular docking with anthocyanins and related compounds have shown that BGL has a preference for anthocyanin-D-arabinosides, followed by D-glucosides and then D-galactosides, a pattern observed in other plant β-D-glucosidases [57]. Additionally, BGL shares a high sequence identity (60.21%) with rice β-glucosidase Os3BGlu6, a member of GH1, which exhibits hydrolytic activity with β-D-glucosides [59]. This implies that BGL can act on β-glucosides, including oligosaccharides.

Docking results showed interactions with glutamic or aspartic acid residues. In the BGL-cyanidin-3-arabinoside complex, the substrate was deep in the active site pocket, forming polar contacts with Gln88, Asn234, Glu451, His377, and Gly453. The catalytic nucleophile Glu451 was deprotonated, while Glu235 acted as the acid/base catalyst, confirming their roles in the enzyme’s mechanism.

## 4. Materials and Methods

### 4.1. Plant Materials

Cranberry samples were collected in November 2023 from small growers in the VII (Maule) and VIII (Biobío) regions of Chile, areas renowned for their rich agricultural heritage. Following harvest, the berries were immediately flash-frozen with liquid nitrogen to preserve their biochemical integrity. They were then transported to the laboratory under controlled conditions. Upon arrival, to ensure the retention of their phytochemical properties, the cranberry samples were stored at −80 °C, awaiting subsequent analysis.

### 4.2. Sample Preparation and UPLC-MS/MS Analysis

The sample preparation and UPLC analysis were carried out in accordance with methodologies reported in the literature [60]. In brief, 100 mg of lyophilized cranberry was placed into a new tube and extracted with 1 mL of a methanol solution, (methanol/water/formic acid = 70:30:1, V/V/V). The samples underwent vigorous vortexing for 1 min and sonication for 20 min, followed by centrifugation at 2000× *g* for 10 min, with the process repeated for double extraction. The combined supernatants were subsequently lyophilized. For LC-MS/MS analysis, the dried extracts were reconstituted in 200 µL of methanol, and 2 µL aliquots of these solutions was injected into a Waters HSS T3 column (2.1 × 50 mm, 1.8 µm) housed within a Vanquish UPLC system. The system was interfaced with a Q Exactive mass spectrometer operating in positive ion mode. The LC separation employed a mobile phase consisting of 0.1% formic acid in water (A) and 0.1% formic acid in acetonitrile (B), utilizing a gradient elution profile: 0–1 min at 5% B, 1–6 min increasing from 5% to 30% B, 6–7 min from 30% to 95% B, held at 95% B for 7–8 min, then returning to 5% B for 8–8.1 min, and finally re-equilibrating at 5% B for 8.1–10 min. The flow rate was maintained at 0.3 mL/min at a column temperature of 40 °C [61,62].

### 4.3. Template Identification and Protein Modeling

The amino acid sequences used to model the cranberry enzymes were from different species: *Rhododendron simsii* (UniProtKB code: A0A834FUZ7) for BGL, *Arabidopsis thaliana* (UniProtKB code: P43311) for POD, and *Vitis vinifera* (PDB code: 1QGJ) for PPO. These sequences were compared against the *Vaccinium* database (www.vaccinium.org accessed on 1 January 2024) using Blastp, focusing on the *Ben Lear* (GDV21001) and *Stevens* (GDV20001) cultivars of cranberry (*Vaccinium macrocarpon*).

Protein modeling was subsequently carried out using the AlphaFold2 server, an artificial intelligence system by DeepMind that predicts protein three-dimensional structures from amino acid sequences with confidence intervals up to 95% [63]. Out of five three-dimensional structural predictions generated, the model chosen for further analysis was determined by superior performance metrics, including the highest predicted Local Distance Difference Test (pLDDT) scores and prediction quality metrics (pTM). These evaluations ensure the selection of a model that closely mirrors the native protein structure, thus enhancing the reliability of subsequent experimental simulations.

### 4.4. Anthocyanase Model Refinement

Molecular dynamics simulations were performed using GROMACS (version 2023.1). The three-dimensional structure models of cranberry anthocyanase, POD, and PPO were selected based on the highest pLDDT scores (range 0–100) and pTM scores (above 0.5) provided by AlphaFold2. To mimic a realistic biological environment, an orthorhombic box was constructed around the 3D model, incorporating a predefined SPC216 water model, which accurately simulates the hydration of the protein. To further stabilize the system, appropriate ions were added, balancing the net charge and replicating physiological ionic conditions. Subsequent energy minimization was conducted to relax the protein structure and remove any steric clashes or unrealistic bond lengths and angles. This step was crucial to ensure the structural integrity and realism of the simulated protein. The system then underwent a careful equilibration process, where both temperature and pressure were methodically stabilized, employing algorithms like the Berendsen thermostat and Parrinello–Rahman barostat for accurate control. The production phase of the simulation extended over a 100 ns time frame, capturing the dynamic behavior of the anthocyanase protein under these conditions. Throughout this phase, the final coordinates of the system were recorded every 30 picoseconds, allowing for a detailed analysis of the protein’s movements and interactions. The refined model, validated and stabilized through this rigorous simulation process, was subsequently employed in molecular docking studies.

### 4.5. Molecular Docking

Molecular docking simulations were carried out employing AutoDock Vina [64] to investigate the interactions among the cranberry enzymes under study—anthocyanase, polyphenol oxidase, and peroxidase—and the anthocyanins and related compounds identified in this research. These analyses also encompassed enzyme-specific substrates to comprehensively understand the enzymatic activity. Ligand structures were optimized using the Avogadro software (version 1.2.0) [65]. The protonation states of the protein and ligands were adjusted to pH 2.4 [66,67].

The receptor grid was located at the anthocyanase binding site, with grid dimensions centered at specified X, Y, and Z coordinates for each enzyme: BGL (center x: 41.593, center y: 45.952, center z: 52.727), POD (center x: 43.669, center y: 41.122, center z: 43.215), and PPO (center x: 48.652, center y: 49.453, center z: 51.249), respectively.

The most stable docking orientation was identified based on binding affinity scores and hydrogen bond interactions with the binding site through visual inspection [23]. PyMol was utilized to generate 3D graphical visualizations of anthocyanase and protein–ligand complexes.

## 5. Conclusions

The enzymatic degradation pathways proposed for anthocyanins in cranberries highlight significant implications for both plant physiology and food science. The identification of key interacting residues provides a foundation for developing inhibitors to preserve anthocyanin stability, thereby enhancing the nutritional quality and shelf life of cranberry products. Future research should focus on validating these computational findings through in vitro and in vivo assays to elucidate the precise biochemical mechanisms underlying anthocyanin degradation. Additionally, exploring the environmental factors influencing enzyme activity could offer strategies to mitigate anthocyanin loss during fruit processing and storage. These findings can be applied in the food industry to develop advanced preservation techniques, such as the use of specific enzyme inhibitors, to improve the stability and longevity of products containing anthocyanins. By integrating these strategies, the food industry can enhance the quality and commercial value of a wide range of anthocyanin-rich products.

## Figures and Tables

**Table 1:** Identification of anthocyanins and related compounds in cranberry fruits by UPLC-MS/MS with corresponding retention times (t_R_) and peak areas (% of total), mean concentrations, and standard deviations (SD). Samples were analyzed in duplicate.

Peak	Compound Name	Cas	Molecular Formula	Molecular Weight (g/mol)	t_R_ (min)	Mass-to-Charge Ratios (*m*/*z*)	Conc. (mg/100 g dm) ± SD
1	Cyanidin	528-58-5	C_15_H_11_ClO_6_	322.69	5.450	287.06	0.005 ± 0.000
2	Cyanidin-3-galactoside	27661-36-5	C_21_H_21_ClO_11_	484.8	3.960	449.11	27.687 ± 0.140
3	Delphinidin	528-53-0	C_15_H_11_ClO_7_	338.69	4.840	303.05	0.025 ± 0.000
4	Procyanidin B4	29106-51-2	C_30_H_26_O_12_	578.5	5.380	579.15	0.277 ± 0.003
5	Procyanidin B2	29106-49-8	C_30_H_26_O_12_	578.5	5.500	579.15	2.034 ± 0.020
6	Petunidin	1429-30-7	C_16_H_13_ClO_7_	352.72	5.620	317.07	0.003 ± 0.000
7	Pelargonidin	134-04-3	C_15_H_11_ClO_5_	306.70	6.070	271.06	0.002 ± 0.000
8	Peonidin	134-01-0	C_16_H_13_ClO_6_	336.72	6.230	301.07	0.004 ± 0.000
9	Malvidin	643-84-5	C_17_H_15_ClO_7_	366.7	6.250	331.08	0.001 ± 0.000
10	Rutin	153-18-4	C_27_H_30_O_16_	610.5	6.780	611.16	0.018 ± 0.000
11	Luteolin	491-70-3	C_15_H_10_O_6_	286.24	8.170	287.06	0.007 ± 0.000
12	Quercetin	117-39-5	C_15_H_10_O_7_	302.23	8.180	303.05	0.452 ± 0.005
13	Kaempferol	520-18-3	C_15_H_10_O_6_	286.24	8.350	287.06	0.008 ± 0.000
14	Isorhamnetin	480-19-3	C_16_H_12_O_7_	316.26	8.360	317.07	0.070 ± 0.001

**Table 2:** Binding affinity energies calculated from molecular docking simulations between β-glucosidase (BGL), peroxidase (POD), polyphenol oxidase (PPO), and the anthocyanins and related compounds found in cranberry. Common ligands include sucrose (SUC) for BGL, Epicatechin (EPC) for POD, and 3,4-Dihydroxyphenylacetic acid (3,4-DHPA) for PPO.

Ligands	Abbreviation	BGL-Binding Energy (kcal/mol)	BGL-Interacting Residues	POD-Binding Energy (kcal/mol)	POD-Interacting Residues	PPO-Binding Energy (kcal/mol)	**PPO-Interacting Residues**
Rutin	RUTIN	−10.1	His189, Glu451, Gly453	−8.9	Arg53, His57, Asn147, Lys187, Asp225	−3.8	Leu424
Cyanidin-3-arabinoside	C3-ARA	−9.3	Gln88, Asn234, Glu451, His377, Gly453	−8.3	Asn147, Ser85, Pro81, His57, Asp225, Arg53	−7.6	Lys502, Asp319, Asn533, His330, Val534
Delphinidin-3-O-arabinoside	D3-O-ARA	−9.3	Gln88, His189, Asn234, Glu242, Lys308, Glu451, Trp501	−8.5	Arg53, His57, Asn147, Lys187	−7.6	Glu198, Asp319, His330, Lys502
Delphinidin 3-O-glucoside	D3-O-GLU	−9.2	Gln88, His189, Asn234, Gly238, Asp306, Lys308, Trp501, Glu508	−8.2	His57, Pro81, Leu83, Ser85, Lys187	−5.5	Cys180, Glu198, Asp319, His330, Ser548, Cu2
Cyanidin-3-glucoside	C3-GLU	−9.2	Gln88, His189, Glu235, Glu451, His377	−8.1	Ala189, His57, Asn147, Arg53	−5.8	Asp195, Val534, Asn327, Val532, Glu326
Pelargonidin 3-galactoside ion	PEL3-GAL-ION	−9.1	His189, Glu235, Gly453	−8.1	Arg53, His57, Pro151, Lys187, Asp225	−6.5	Asn327, His536, His538, His540
Pelargonidin 3-Galactoside	PEL3-GAL	−9.1	His189, Glu235	−8.1	Arg53, His57, Lys187	−6.5	Glu198, Asn327, His536, His538, His540
Quercetin-3-O-galactoside-7-O-glucoside	QUE3-GAL-7-GLU	−9.1	His189, Asn234, Arg237, Glu242, Lys308	−8.2	Arg53, His57, Leu83, Glu155, Asn159, Lys163	−3.8	Tyr421
Petunidin 3-glucoside	PET3-GLU	−8.9	Ser85, Gln88, His189, Glu242, Lys308, Trp501	−8.1	Arg53, His57, Pro81, Ser85, Pro151, Asp225	−5.0	Arg369, Ser372, Glu415
kaempferol 3-O-arabinoside	K3-ARA	−8.9	Ser85, Gln88	−8.3	Arg53, His57, Pro151, Asp225	−6.1	His330, Val534, CU2
Malvidin-3- Arabinoside	M3-ARA	−8.8	Glu235, Arg237, Lys308, Tyr310, Tyr378	−7.9	Arg53, His57, Ala80, Asp225	−6.9	His330, Val534
Petunidin 3-arabinoside	PET3-ARA	−8.8	Ser85, Gln88, His189, Asn234, Asp306, Lys308, Tyr378, Glu451, Trp501	−8.1	Arg53, His57, Ala80, Pro81	−7.2	Glu198, His330, Val534
Pelargonidin 3-arabinoside	PEL-3ARA	−8.8	Gly238, Glu242, Lys308, Glu451	−8.2	Arg53, His57	−7.6	Glu198, His538, His540
Pelargonidin 3-glucoside	PEL3-GLU	−8.8	Gln88, His189, Gly453	−8.2	Arg53, His57, Asn84, Ala88	−5.6	Asn327, His536, His540, Ser548
Quercetin 3-O-arabinoside	QUE3-ARA	−8.8	Gln88, Arg145	−8.1	Arg53, His57, Asp225	−7.5	Asp195, His330, Val534, Cu2
Peonidin- 3- galactoside	P3-GAL	−8.7	His189, Glu451, Gly453	−8.0	Arg53, His57, Lys187	−6.4	Asn327, Val534, His330
Peonidin -3- glucoside	P3-GLU	−8.7	Lys308, Asp306, Glu242, Gly238, Gln88, Trp501	−8.2	Asp225, Ser85, Pro81, His57, Arg53, Pro151	−5.3	Ser414, Ser372, Tyr421, Arg369
Petunidin 3-galactoside	PET3-GAL	−8.7	Gln88, His189, Asn234, Lys308, Glu451, Trp501	−7.9	Arg53, His57, Ala189, Asp225	−5.1	Asp319, Glu326, Asn327, Val534
Kaempferol 3-O-glucoside	K3-GLU	−8.7	Gln88, Asn234, Gly238, Glu242, Glu508	−8.3	Arg53, His57, Pro81, Ser85, Pro151, Asp225	−5.4	Cys180, Asn327, His536, His540, Ser548
Peonidin- 3- arabinoside	P3-ARA	−8.6	Asp306, Lys308, Glu451, Trp501, Gln88, Ser85	−8.1	Ser85, Pro81, Arg53, His57	−7.3	Lys502, Asp319, Asn533, Val534, His330
Isorhamnetin 3-O-galactoside	ISO3-GAL	−8.6	Gln88, Glu235, Gln249, Glu451, Trp501, Trp509	−8.1	Arg53, His57, Asp225	−5.3	Arg369, Leu424
Cyanidin-3-galactoside	C3-GAL	−8.5	Trp509, Gln88, Trp501	−8.0	Asp225, Leu83, His57, Arg53	−5.6	Asp195, His197, Ser331
Malvidin-3- galactoside	M3-GAL	−8.5	Tyr378, Lys308, Trp509, Asn234, His189	−7.7	Arg53, His57, Asp225	−5.6	Glu198, Glu326, Asn327, His330, Val534
Delphinidin 3-O-galactoside	D3-GAL	−8.4	Gln88, His189, Asn234, Ile248, Asp306, Lys308, Glu451	−8.0	Arg53, His57, Pro81, Leu83, Ser85	−6.1	Asn327, Ser331, His536
Isorhamnetin 3-O-glucoside	ISO3-GLU	−8.3	Gln88, Trp501, Trp509	−8.1	Arg53, His57, Ser85, Pro151, Asp225	−4.7	Tyr421, Leu424
Malvidin-3- glucoside	M3-GLU	−8.1	His189, Asn234, Glu242, Lys308, Tyr378, Glu451	−8.0	Arg53, His57	−4.9	Arg369, Gln422, Asn423 Leu424
Luteolin	LUTEOLIN	−7.8	His377, Gly453, Glu508, Trp509	−8.1	Arg53, His57, Lys187	−8.6	Asn327, Val532, His538
Positive Control							
Sucrose	SUC	−6.8	Glu451, Glu242, Trp509, Gln88	-		-	-
Epicatechin	EPC	-		−8.3	Ala189, Arg53, His57, Pro151	-	-
3,4-dihydroxyphenylacetic	3,4- DHPA	-		-		−6.6	Glu198, Ala322, Tyr549, Ser530

## Data Availability

The data presented in this study are available on request from the corresponding author.

## Supplementary Materials

The following supporting information can be downloaded at: https://www.mdpi.com/article/10.3390/ijms251910437/s1.
