# Supplementary material for: In Silico Analysis of the Molecular Interaction between Anthocyanase, Peroxidase and Polyphenol Oxidase with Anthocyanins Found in Cranberries
Source: Int J Mol Sci. 2024 Sep 27;25(19):10437. doi: 10.3390/ijms251910437 (PMC11476609; doi:10.3390/ijms251910437)
Supplement: Supplementary file 1 [file ijms-25-10437-s001.zip › ijms-3140828-supplementary.pdf]

**Figure S1:** HPLC Chromatograms and Quantitative Summary Report of Anthocyanins and Related Compounds Identified in Cranberries.

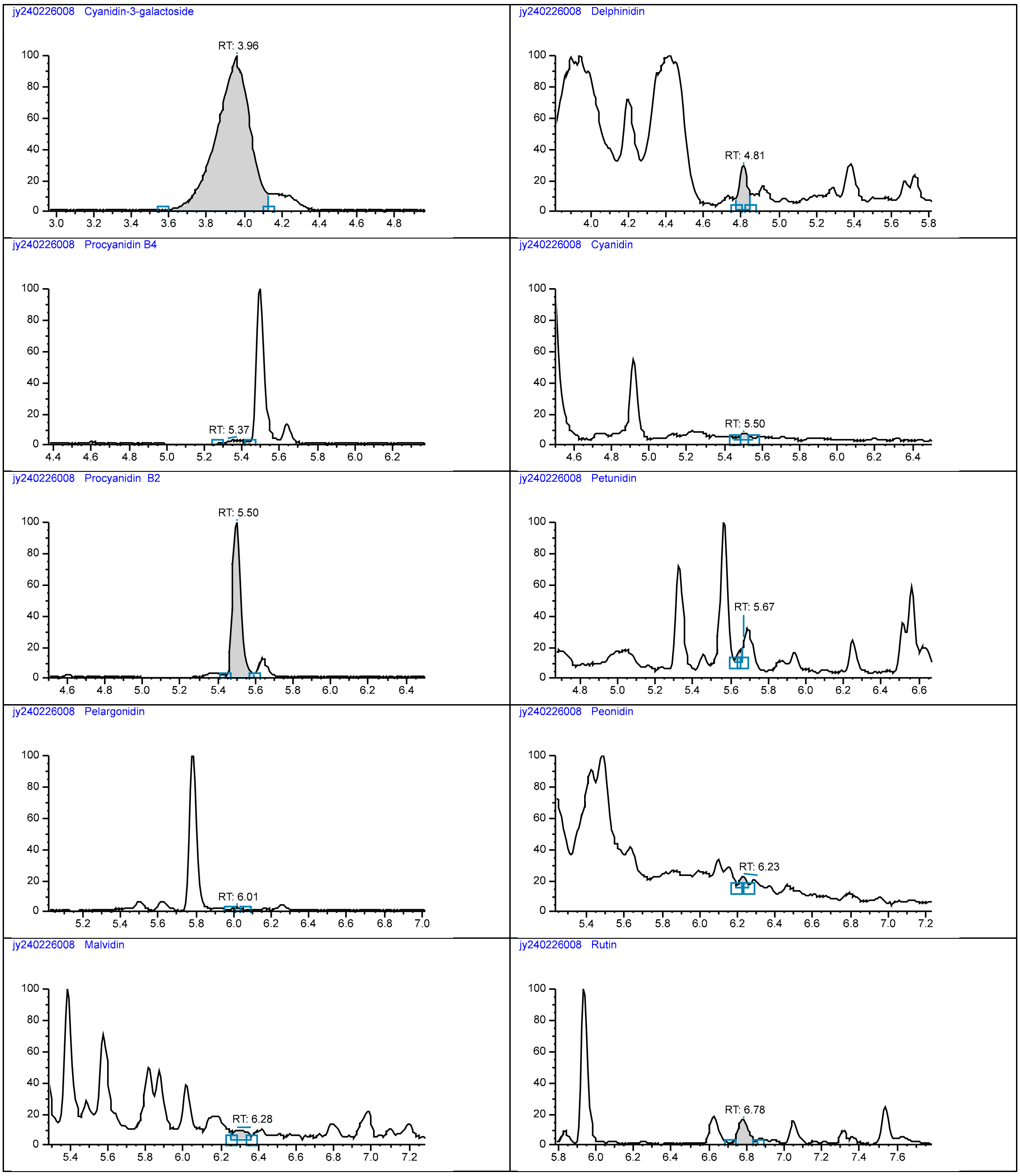

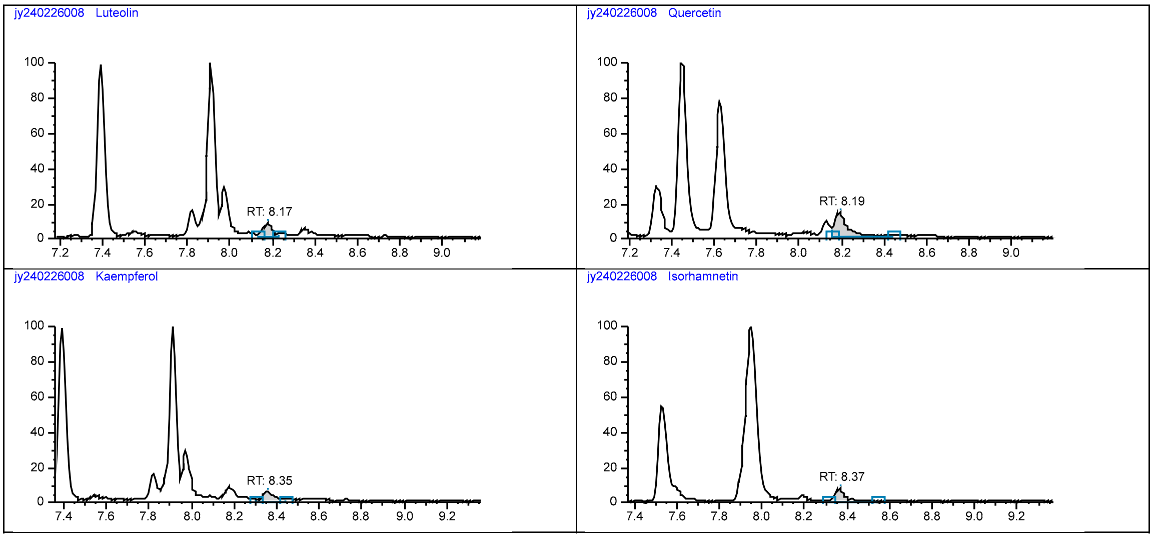


## Quantitation Report - Target Compounds_ Cranberries

| Sample | Compound Name | Expected Mass (m/z) | Detected Mass | m/z Delta | m/z Delta Units | RT | Peak Height | Response | Conc. (mg/100gdm) | SD |
|---|---|---|---|---|---|---|---|---|---|---|
| 1 | Cyanidin-3-galactoside | 449,1078 | 449,11 | 0,020 | ePPM | 3,958 | 598657462 | 7241838276 | 27.687 | ± 0.140 |
| 2 | Delphinidin | 303,0499 | 303,05 | -1,622 | ePPM | 4,809 | 212496 | 619868 | 0.025 | ± 0.000 |
| 3 | Procyanidin B4 | 579,1497 | 579,15 | -0,599 | ePPM | 5,370 | 1054737 | 6531710 | 0.277 | ± 0.003 |
| 4 | Cyanidin | 287,0550 | 287,05 | -0,805 | ePPM | 5,498 | 175576 | 579560 | 0.005 | ± 0.000 |
| 5 | Procyanidin B2 | 579,1497 | 579,15 | -1,231 | ePPM | 5,498 | 44125706 | 135682217 | 2.034 | ± 0.020 |
| 6 | Petunidin | 317,0656 | 317,07 | -0,089 | ePPM | 5,666 | 131519 | 185068 | 0.003 | ± 0.000 |
| 7 | Pelargonidin | 271,0601 | 271,06 | -0,265 | ePPM | 6,014 | 115479 | 338433 | 0.002 | ± 0.000 |
| 8 | Peonidin | 301,0707 | 301,07 | -0,039 | ePPM | 6,229 | 220998 | 515118 | 0.004 | ± 0.000 |
| 9 | Malvidin | 331,0812 | 331,08 | -0,253 | ePPM | 6,282 | 19586 | 105602 | 0.001 | ± 0.000 |
| 10 | Rutin | 611,1607 | 611,16 | -1,224 | ePPM | 6,780 | 420184 | 1717858 | 0.018 | ± 0.000 |
| 11 | Luteolin | 287,0550 | 287,06 | 0,365 | ePPM | 8,174 | 1000526 | 2822256 | 0.007 | ± 0.000 |
| 12 | Quercetin | 303,0499 | 303,05 | -0,212 | ePPM | 8,192 | 37497681 | 134137077 | 0.452 | ± 0.005 |
| 13 | Kaempferol | 287,0550 | 287,05 | -0,486 | ePPM | 8,355 | 746860 | 2676653 | 0.008 | ± 0.000 |
| 14 | Isorhamnetin | 317,0656 | 317,07 | 0,103 | ePPM | 8,366 | 7773782 | 22871438 | 0.070 | ± 0.001 |
